# Recycled Carbon Fiber-Supported Polyaniline/Manganese Dioxide Prepared via One-Step Electrodeposition for Flexible Supercapacitor Integrated Electrodes

**DOI:** 10.3390/polym10101152

**Published:** 2018-10-16

**Authors:** Xiaoning Wang, Hongli Wei, Wei Du, Xueqin Sun, Litao Kang, Yuping Zhang, Xiangjin Zhao, Fuyi Jiang

**Affiliations:** School of Environment and Material Engineering, Yantai University, Yantai 264005, China; yantaiwxn@163.com (X.W.); ytdxwhl@163.com (H.W.); sxq@ytu.edu.cn (X.S.); kanglitao@ytu.edu.cn (L.K.); zhangyuping306@163.com (Y.Z.); zl2915@ytu.edu.cn (X.Z.); fyjiang@ytu.edu.cn (F.J.)

**Keywords:** recycled carbon fiber, polyaniline, manganese dioxide, flexible electrode, supercapacitor

## Abstract

The exploration of multifunctional electrode materials has been a hotspot for the development of high-performance supercapacitors. We have used carbon fiber plates recovered from construction waste to prepare high-quality flexible carbon fiber materials by pyrolysis of epoxy resin. The as-prepared recycled carbon fiber has a diameter of 8 μm and is the perfect substrate material for flexible electrode materials. Furthermore, polyaniline and manganese dioxide are uniformly deposited on the recycled carbon fiber by one-step electrodeposition to form an active film. The recycled carbon fiber/polyaniline/MnO_2_ composite shows an excellent specific capacitance of 475.1 F·g^−1^ and capacitance retention of 86.1% after 5000 GCD cycles at 1 A·g^−1^ in 1 M Na_2_SO_4_ electrolyte. The composites optimized for electrodeposition time have more electroactive sites, faster ions and electron transfer, structural stability and higher conductivity, endowing the composites promising application prospect.

## 1. Introduction

With the rapid growth of the population and the global economy, the consumption of various fossil fuels has increased significantly, resulting in a serious negative impact on the environment, such as the greenhouse effect and acid rain. Stimulated by the above environmental problems and the growing demand for grid energy storage and consumer electronics, there is an urgent demand for new and green energy storage systems [1,2,3,4,5]. The development of various new electrochemical energy storage devices (EES) such as supercapacitors (SCs), lithium ion batteries, and biofuel cells is in full swing. Among them, with the rise of new energy vehicles, SCs have become one of the most promising energy storage devices, owing to their excellent power output (>10 kW·kg^−1^) and long cycle life (>100,000 cycles) [3].

Nowadays, flexible electrode materials are attracting more and more attention due to the development of a variety of emerging consumer electronic devices, such as portable and wearable electronic devices, smart watches, integrated displays and flexible mobile phones [6]. Compared with traditional two-dimensional (2D) planar structural materials, one-dimensional (1D) fibrous materials (such as carbon nanotubes, graphene fibers, carbon fibers, etc.) are widely used in the preparation of flexible electrode materials due to their light weight, good electrical conductivity and high flexibility [7,8,9]. Owing to the unique ultra-high mechanical properties of carbon fibers (CFs), they are a perfect choice for acting as flexible electrode substrates. It is estimated that by 2020, global carbon fiber production will reach 112,000 tons. Although it has a high commercial output, the unit price of high-quality CFs is basically maintained at about $60 kg^−1^, and the high cost has become a stumbling block in the application of flexible electrodes [10]. Carbon fiber-reinforced polymer composites have been widely used in aerospace, high-end sports equipment and the automotive industry due to their ultra-low weight, corrosion resistance, low thermal expansion properties and ultra-high strength. Especially in the construction industry, carbon fiber-reinforced epoxy resin matrix composite is favored for its high tensile strength, corrosion resistance and impact resistance. On the other hand, these composite materials will eventually be converted into intractable construction waste. These construction wastes not only occupy a large amount of land resources, but also cause inevitable environmental pollution, such as air and water pollution. In view of cost reduction and environmental friendliness, we chose to recycle CFs from construction waste to achieve the goal of turning waste into treasure.

It is key to improving the performance of SCs by anchoring the appropriate active materials on CFs flexible substrates. Polyaniline (PANI) is becoming the most promising conductive polymer electrode material for SCs due to its large specific capacitance (400–750 F·g^−1^), high conductivity, easy synthesis, low cost and sensitive reversibility between redox states [11,12]. However, the expansion, shrinkage and irreversible degradation of PANI during charging/discharging degrade its mechanical properties and affect its electrochemical performance [13]. One of the effective ways to improve the stability of PANI is to prepare a multi-component hybrid material by doping PANI with some nanomaterials such as metal oxides and carbon materials [14]. Compared to pure PANI, multi-component materials show more stable and excellent electrochemical performance. The hybridization process of multi-component materials typically requires the preparation of two or more reaction steps, which is obviously time consuming and uneconomical. It is worth mentioning that the one-step electrodeposition method was chosen because of its simplicity, efficiency and low cost. MnO_2_ is a typical pseudocapacitor material similar to PANI, which can be more easily reduced and has the characteristics of low cost and high theoretical specific capacitance (1370 F·g^−1^) [9]. During the electrodeposition process, the unique redox activity of MnO_2_ may affect its multi-hybrid properties with PANI hybridization, and may also act as electron acceptors to effectively stabilize the structure of PANI [15,16].

In this work, we prepared recycled carbon fiber from carbon fiber reinforced epoxy resin matrix composite through high-temperature pyrolysis. On this basis, the recycled carbon fiber-supported PANI/MnO_2_ flexible integrated electrode was successfully prepared by a one-step electrodeposition technique. It is shown that this ternary composite is not a simple multi-phase superposition process. When electropolymerization of aniline and electrodeposition of MnO_2_ occur simultaneously, the formed MnO_2_ can act as an oxidant to further initiate chemical polymerization of aniline, resulting in a fluffy structure and rapid formation of the hybrid film. The donor-receptor interaction between PANI and MnO_2_ enhances electron transport due to the complementary conductivity of the two components. The recycled carbon fiber/PANI/MnO_2_ composite shows a large specific capacitance of 475.1 F·g^−1^ at 1 A·g^−1^ in 1 M Na_2_SO_4_ electrolyte and excellent capacitance retention of 86.1% after 5000 GCD cycles.

## 2. Experimental Section

### 2.1. Materials

Carbon fiber plates (CFP) collected from construction waste are composites formed by the infiltration and hardening of carbon fibers in epoxy resins (originally produced by Shandong Yingteli New Material Co., Ltd., Jining, China). All chemical reagents, including Aniline, Mn(CH_3_COO)_2_·4H_2_O, Na_2_SO_4_ are analytical grade without further purification.

### 2.2. Recovery of Carbon Fiber from CFP

Firstly, the CFP was cut into a rectangular shape (1 cm × 2 cm) and placed in a crucible, and then the epoxy resin was pyrolyzed at a high temperature in a muffle furnace under an air atmosphere at 450 °C for 2 h. After naturally cooling to room temperature, the as-obtained carbon fiber was soaked in 1 M HCl and sonicated for 30 min to remove inorganic impurities on the fiber surface, then washed with deionized water and ethanol, and thoroughly dried in an oven at 60 °C. The prepared sample was recycled carbon fiber (RCF).

### 2.3. Synthesis of RCF/PANI, RCF/MnO_2_ and RCF/PANI/MnO_2_

**Figure 1:**
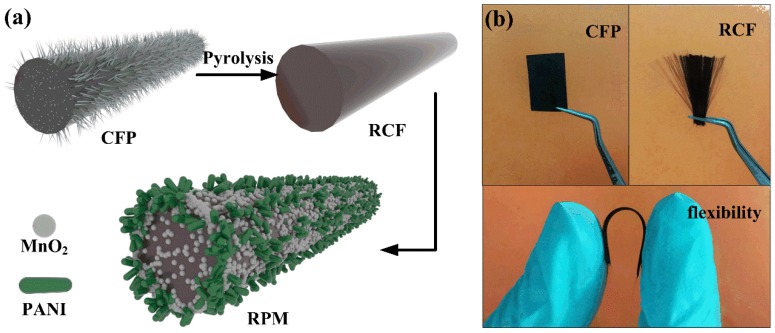
(**a**) Schematic illustration of the fabrication process for the RPM electrode. (**b**) Photographs of carbon fiber plates (CFP) and recycled carbon fiber (RCF).

The RCF-supported PANI/MnO_2_ flexible integrated electrode was prepared by one-step electrodeposition. The anode electrodeposition method was used. The saturated calomel (SCE), platinum foil and RCF were used as the reference electrode, the counter electrode and the working electrode, respectively. The anodic electrodeposition of PANI/MnO_2_ was performed in aqueous solution containing 0.1 M Mn(CH_3_COO)_2_, 0.1 M Na_2_SO_4_, 0.5 M H_2_SO_4_ and 0.5 M aniline at a deposition voltage of 0.8 V for 5, 7.5 and 10 min, labeled RPM-5, RPM-7.5, and RPM-10, respectively. The deposited RCF was washed with deionized water and ethanol and dried at 60 °C. In the control experiment, Mn(CH_3_COO)_2_ and aniline were absent from the electrolyte, and electrodeposition was performed for 7.5 min to obtain RCF/PANI and RCF/MnO_2_ composite samples, which were named RP-7.5 and RM-7.5, respectively. The flow chart of the RPM electrode synthesis process and photographs of CFP and RCF are illustrated in Figure 1.

### 2.4. Materials Characterization

Scanning electron microscopy (SEM, Hitachi S-4800, Tokyo, Japan) was used to characterize the structures and morphological analysis of the as-prepared sample. X-ray diffraction (XRD) measurement was carried out by an X-ray diffractometer (Shimadzu-7000X, Tokyo, Japan) with Cu Kα radiation operated at 40 kV and 30 mA in the range of 2θ = 10–80°. Thermogravimetry (TG) tests were carried out by thermogravimeter in air atmosphere (Netzsch STA449 F3, Selb, Germany). Pore structure of samples was characterized by N_2_ adsorption/desorption analyzer (ASAP 2020, Norcross, GA, USA). Fourier transformation infrared spectra (FTIR, Shimadzu IR Prestige-21, Tokyo, Japan) of the samples were measured. Raman spectra were collected on Raman spectrometer (Thermo Nicolet NEXUS 670, Waltham, MA, USA) with a 514 nm laser excitation. 

### 2.5. Electrochemical Measurements

The electrochemical measurements of the prepared electrodes were carried out in three-electrode systems with 1 M Na_2_SO_4_ as electrolyte. Cyclic voltammetry (CV), galvanostatic charge-discharge (GCD), and electrochemical impedance spectroscopy (EIS) measurements were conducted by employing an electrochemical workstation (CHI 660E, Shanghai, China). The saturated calomel (SCE), platinum foil and samples were used as the reference electrode, the counter electrode and the working electrode, respectively. CV and GCD tests were performed in the potential range of −0.2–0.8 V. EIS measurements were performed with a frequency range of 100 kHz to 0.01 Hz with an AC amplitude of 5 mV at open circuit voltage. The specific capacitances of samples were calculated based on GCD curves according to the following Equation (1):

(1) $$C_{s}=\frac{I\times \Delta t}{m\times \Delta V}$$

where *I* (A) is the constant discharge current, Δt (s) is the discharge time, *m* (g) is the mass of active material and ΔV (V) is the potential window.

## 3. Results and Discussion

### 3.1. Characterization of Materials

**Figure 2:**
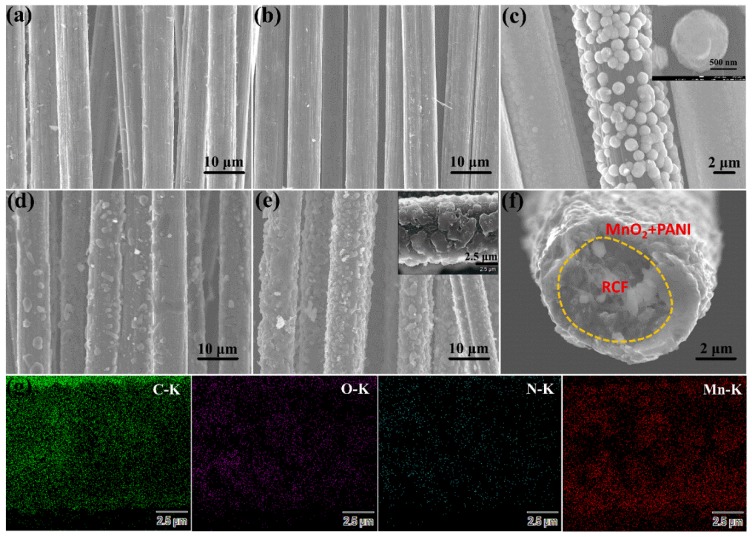
The scanning electron microscopy (SEM) images of (**a**) CFP, (**b**) RCF, (**c**) RM-7.5, (**d**) RP-7.5, (**e**,**f**) RPM-7.5 composite, and (**g**) energy dispersive spectroscopy (EDS) elemental mapping of C, N, O and Mn on the inset of Figure 2e.

The microscopic morphologies of the different materials can be observed by SEM images in Figure 2. As shown in Figure 2a,b, there are significant differences between untreated CFP and pyrolyzed RCF. The surface of the CFP contains distinct and tiny burr-like impurities, which may be residual epoxy resins and other organic components. The organic polymer materials on the surface of CFs are mostly poor conductors, which reduce the conductivity of CFs. However, the RCF surface in Figure 2b exhibits a smoother morphology, which can be due to the removal of the epoxy resin after high-temperature pyrolysis and pickling. The smooth and highly conductive RCF surface is more likely to combine with other components to provide an excellent substrate for the synthesis of high-performance capacitive materials. The surface of RM-7.5 (in Figure 2c) exhibits a granular morphology with a diameter of about 1 μm due to the deposition of MnO_2_. There is an inevitable difference in the amount of deposition of each carbon fiber, so that excess manganese dioxide particles form a shell covering the surface of the RCF. As shown in Figure 2d, the PANI presents irregular rod-like particles dispersed on the surface of the RCF. It can be clearly seen from Figure 2e that the electrodeposition material on the surface of the RCF is significantly increased compared to the RP&RM-7.5, which further proves that the recombination of the two components is not a simple hybridization process. When electropolymerization of aniline and electrodeposition of MnO_2_ occur simultaneously, the formed MnO_2_ can act as an oxidant to further initiate chemical polymerization of aniline, resulting in a rapidly formed mixed film. Figure 2f shows the SEM image of the high-magnification cross-section of RPM-7.5. The RCF surface is covered with the MnO_2_ and PANI composite film with a thickness of about 1 μm to form a stable shell structure. Moreover, energy dispersive spectroscopy (EDS) elemental mapping images of the RPM-7.5 are displayed in Figure 2g. Here, the O, N and Mn elements are uniformly distributed in the active material, and the C element is uniformly distributed on the RCF. 

**Figure 3:**
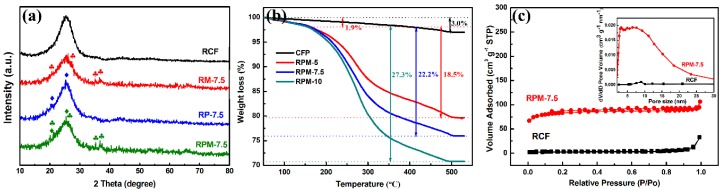
(**a**) The X-ray diffraction (XRD) patterns of RCF and RM-7.5, RP-7.5, RPM-7.5 composites. (**b**) Thermogravimetry (TG) of the CFP, RPM-5, 7.5, 10 composites in air atmosphere. (**c**) Nitrogen adsorption-desorption isotherm and BJH pore-size distribution.

The crystal structure of the different materials was determined by XRD analysis. The XRD patterns of RCF and RM-7.5, RP-7.5, RPM-7.5 composites are shown in Figure 3a. The diffraction peak of the RCF after pyrolysis appeared at 2θ = 25° and 45°. The peak appearing at 25° may be due to the presence of the (002) graphite microcrystal plane [17], and the weak peak appearing around 45° may be attributed to the diffusion scattering of the (010) disordered carbon structure [18]. The typical diffraction peaks of PANI in RP-7.5 are locked at 25.5° and 20.5°, corresponding to the (200) and (020) crystal planes of PANI in an emeraldine salt form, respectively [19]. The four diffraction peaks of RM-7.5 at 21.3°, 26.8°, 34.1°, and 36.8° correspond to the (200), (101), (301), and (210) planes of ramsdellite MnO_2_ (JCPDS 42-1316). The XRD pattern of RPM-7.5 contains the above diffraction peaks, which proves that the PANI and MnO_2_ composite film is successfully synthesized. The TG tests were used to analyze the component content of the composite under air atmosphere in Figure 3b. The CFP reached a steady state around 450 °C, and the weight loss at this time was stable at about 3%, which proved that the RCF content in the composite reached 97%. All of the RPM composites exhibit a slight weight loss (1.9%) below 180 °C due to moisture volatilization and initial decomposition of PANI. At 180–480 °C, the weight loss is greatly reduced, which can be attributed to the complete oxidation of PANI, leading to its complete combustion. At 480 °C, the TG curves of the three materials remained essentially stable, with the remaining masses being 79.6% (RPM-5), 75.9% (RPM-7.5) and 70.8% (RPM-10), respectively. The conversion temperature of MnO_2_ is higher than 530 °C, which means that the loss mass contribution after 180 °C is derived from the complete oxidation of PANI, so the PANI content of the three materials is 18.5%, 22.2% and 27.3%, respectively. The structural characteristics of the RCF and RPM-7.5 evaluated by N_2_ adsorption/desorption isotherm and pore-size distribution analysis are as shown in Figure 3c. The N_2_ isotherm of the RPM-7.5 sample shows a significant hysteresis loop at a relative pressure of 0.2–0.9, indicating the presence of porous structures. The specific surface areas of the RCF and RPM-7.5 were calculated to be 16 and 208 m^2^·g^−1^, respectively. This indicates that the specific surface area of the RPM-7.5 was significantly increased by electrodepositing PANI and MnO_2_ on the RCF surface. The pore size distribution curves (the inset of Figure 3c) were calculated using the Barrett-Joyner-Halenda (BJH) model for the adsorption isotherm. It can be seen that the pore structure of RPM-7.5 is more abundant, resulting in a higher specific surface area.

**Figure 4:**
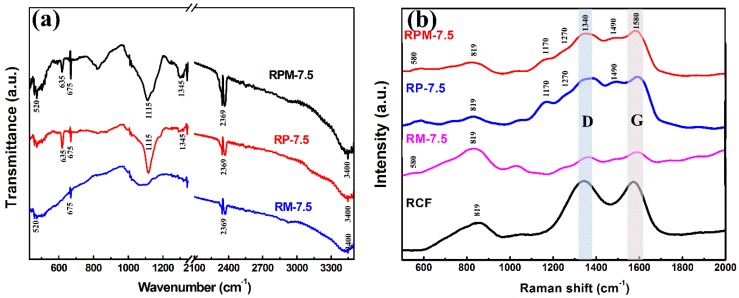
(**a**) Fourier transformation infrared spectra (FTIR) of the RP-7.5, RM-7.5 and RPM-7.5 composites. (**b**) Raman spectra of the RCF and RM-7.5, RP-7.5, RPM-7.5.

The chemical structures of the different samples were investigated using FTIR and Raman spectra, respectively, as shown in Figure 4. The absorption broad peaks present in all three curves in Figure 4a at 3400 cm^−1^ can be attributed to the H–O–H bending of the absorbed H_2_O molecules, and the narrow peaks at 675 and 2369 cm^−1^ can be ascribed to the P–C and P–H stretching vibrations, respectively [20]. The FTIR spectrum of RP-7.5 indicates that PANI has a high-intensity vibration peak in the range of 500–1350 cm^−1^, confirming the high content of functional groups in PANI. The absorption peaks at 1115 and 1345 cm^−1^ are attributed to N=Q=N stretching (Q is the quinonoid ring) and C–N stretching vibrations for the benzenoid ring [21]. The absorption peak at 520 cm^−1^ of the RM-7.5 curve can be assigned to Mn–O vibration, which proves the presence of MnO_2_ [22]. RPM-7.5 contains a multi-component composite of MnO_2_ and PANI, so the spectrum contains all of the above absorption peaks and more functional groups. As shown in Figure 4b, in the Raman spectra of all samples, two peaks around 1340 and 1580 cm^−1^ are assigned to the D and G band of the carbon fiber, respectively. The Raman D peak is attributed to disordered graphite or crystal defects, and the Raman G peak is an ideal graphite lattice vibration mode with E_2g_ symmetry [23]. In the Raman spectrum of RCF, it was noted that there is a distinct peak at 819 cm^−1^ C–H out-of-plane bending of the quinoid ring. The peak of the C–H out-of-plane bending of the quinoid ring was present in all samples, which may be attributed to the residue of the organic polymer on the RCF surface during pyrolysis [3]. In the Raman curves of RPM-7.5 and RM-7.5, the peak at 580 cm^−1^ is related to the Mn–O stretching vibration, indicating the presence of MnO_2_. In the Raman curves of RP-7.5 and RMP-7.5, the characteristic peaks of PANI are overlapped by D\G peaks. The peaks of the two curves at 1170, 1270, 1350, 1490 and 1596 cm^−1^ can be divided into imine deformations, C–H bending, C–N^+^ stretching vibration, C=N stretching vibration of the anthracene ring and C–C stretching of the benzenoid ring, respectively [24].

### 3.2. Electrochemical Measurements

**Figure 5:**
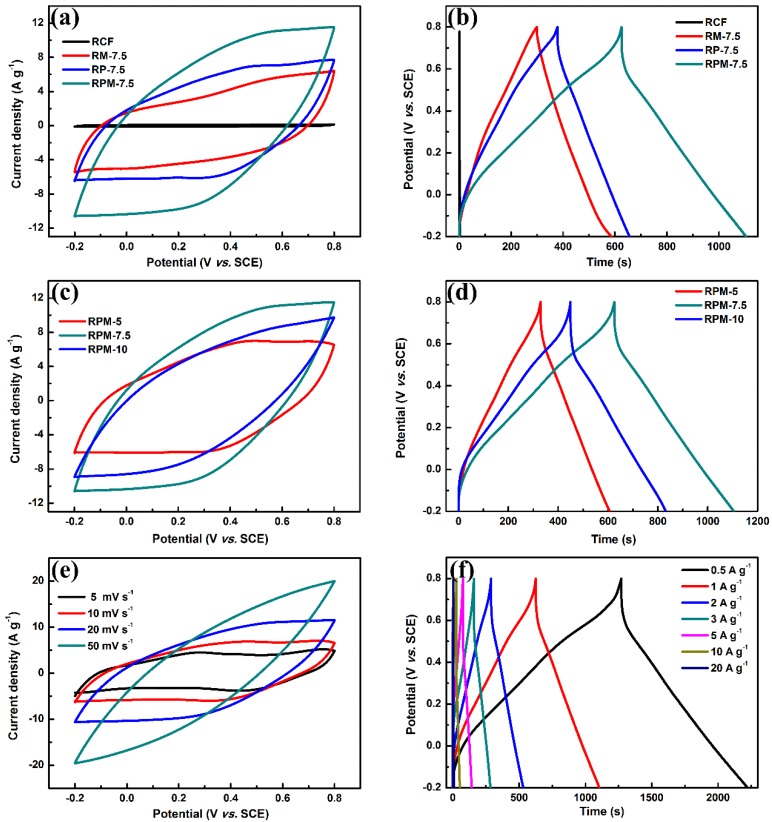
The electrochemical performances of RCF, RM-7.5, RP-7.5, and RPM-5, 7.5, 10: (**a**,**c**) Cyclic voltammetry (CV) curves at a scan rate of 20 mV·s^−1^, and (**b**,**d**) galvanostatic charge-discharge (GCD) curves at a current density of 1 A·g^−1^. (**e**) CV curves at different scan rates, and (**f**) GCD curves at different current densities of RPM-7.5.

The CV curves of the CRF, RM-7.5, RP-7.5 and RPM-7.5 composites were measured at the same scan rate of 20 mV·s^−1^ in 1 M Na_2_SO_4_ electrolyte, as shown in Figure 5a. It can be clearly seen that the shape of the CV curve RM-7.5 is similar to that of RP-7.5. The two curves showed a slight characteristic redox peak at around 0.3 and 0.5 V, while the peak of the curve RM-7.5 was not obvious due to the absence of PANI. The redox peaks of RP-7.5 and RPM-7.5 can be attributed to the redox transition of the PANI between leucoemeraldine/emeraldine and emeraldine/pernigraniline [25]. In addition, the CV curve of RPM-7.5 exhibits a significantly larger integrated area than the other samples, which demonstrates that RPM-7.5 has a stronger active site and ion/electron transport properties, resulting in optimal electrochemical performance. In Figure 5b, according to Equation (1), the specific capacitances of RCF, RM-7.5, RP-7.5 and RPM-7.5 at a current density of 1 A·g^−1^ are 1.8, 288.9, 276.7, 475.1 F·g^−1^, respectively. These results are consistent with the results of the CV curves. The excellent performance of RPM composite may not be a simple hybrid process in a one-step electrodeposition process. When electropolymerization of aniline and electrodeposition of MnO_2_ occur simultaneously, the formed MnO_2_ can act as an oxidant to further initiate chemical polymerization of aniline, resulting in a fluffy structure and rapid formation of the hybrid film. The donor-receptor interaction between PANI and MnO_2_ enhances electron transport due to the complementary conductivity of the two components [26,27]. The different electrodeposition time was used to explore the effect of RPM electrodeposition time on energy storage. The CV and GCD curves are shown in Figure 5c,d. The masses of the active hybrid membranes on the surface of RCF at different electrodeposition times were 4.11 (5 min), 6.14 (7.5 min) and 8.71 mg (10 min), respectively. It can be observed from the CV curve that the integral area reaches the maximum when the electrodeposition time is 7.5 min. The values of IR drop (Figure 5d) also showed slight increases with the increase in deposition time, and were 0.093, 0.118 and 0.157 V, respectively. This indicates that excess active material increases the internal resistance of the electrode as the deposition time increases [28]. The specific capacitances with different deposition times were calculated by GCD curves (Figure 5d), which were 273.9 F·g^−1^ (5 min), 475.1 F·g^−1^ (7.5 min) and 382.8 F·g^−1^ (10 min), respectively. It is obvious that the specific capacitances of the RPM electrodes do not always increase with deposition time. They increase with deposition time from 5 to 7.5 min, which may be attributed to an increase in the active site as the deposited active material mass increases, thereby introducing more pseudocapacitance contribution. However, the specific capacitances decreased significantly as the deposition time increased from 7.5 to 10 min. As the thickness of MnO_2_/PANI increased with increasing electrodeposition time, the low electrical conductivity and the low diffusion constant limited the energy storage performance, which was verified by IR drop and EIS analysis. Figure 5e shows the CV curve of the RPM-7.5 electrode in a potential window of −0.2 to 0.80 V at various scan rates of 5 to 50 mV·s^−1^. As expected, RPM-7.5 showed a slight characteristic redox peak due to the pseudocapacitance contribution of PANI and MnO_2_. Among them, the energy storage mechanism of MnO_2_: MnO_2_ + *M*^+^ +e^−^ ⇌ (MnOO)*M* (where *M* = Li^+^, Na^+^, K^+^) [16]. As the scan rate increases, the gradual distortion of the curve becomes elliptical and shows a significant positive and negative scan volt-ampere charge increase, which may be caused by the internal resistance of the electrode preventing the charge collection of the charge and the diffusion of Na^+^ [29]. As the current density increases from 0.5 to 20 A·g^−1^, the specific capacitances gradually decrease as follows: 493 F·g^−1^ (0.5 A·g^−1^), 475 F·g^−1^ (1 A·g^−1^), 455 F·g^−1^ (2 A·g^−1^), 384 F·g^−1^ (3 A·g^−1^), 330 F·g^−1^ (5 A·g^−1^), 251 F·g^−1^ (10 A·g^−1^), 239 F·g^−1^ (20 A·g^−1^). It is worth noting that RPM-7.5, shown in Table 1, exhibits higher specific capacitance performance compared to other carbon/PANI/MnO_2_ in the previous literature. This may be attributed to the unique interdependence of MnO_2_ nanoparticles and PANI nanorods on the RCF surface for faster electron transport, which compensates for the poor conductivity and low charge-discharge rate of MnO_2_. In addition, hydrogen bond interactions between MnO_2_ and PANI can reduce the electron transfer barrier and increase *C*_sp_ [30].

**Figure 6:**
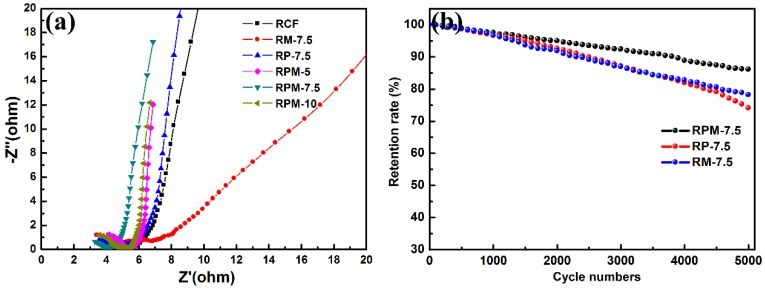
(**a**) Nyquist plots of different samples and (**b**) Cyclestability of RPM-7.5, RP-7.5 and RM-7.5 at a current density of 10 A·g^−1^.

The charge transport and ion diffusion behaviors of different samples were further studied by EIS, and the results are shown in Figure 6a. The value of the real part represents the internal resistance when the imaginary part tends to zero, including the electrolyte resistance, the intrinsic resistance of the RCF/PANI/MnO_2_, and the contact resistance between the interface active material and the current collector. In this work, the intercept shown in the high frequency semicircle represents the polarization or charge transfer resistance of the electrode due to the use of the same electrolyte and current collector during the test [35,36]. RCF has a higher charge transfer resistance in different samples, which can be attributed to the adverse effects of pyrolysis organic residues on the electrical conductivity. It can be noted from the EIS curves that the charge transfer resistance values and the Warburg resistance are both reduced by the introduction of PANI on RCF surface. In addition, RPM-7.5 has a smaller X-axis cross section in the high frequency region and a higher slope in the low frequency region, which means lower equivalent series, charge transfer resistance and higher ion diffusion. This represents the fact that ion insertion and extraction can be achieved more quickly in the electrochemical process, which may be attributed to the fact that the MnO_2_-doped PANI film can enhance electron delocalization and improve electron transport efficiency [37].

Cycle stability is an important reference factor for the quality of supercapacitor electrode materials. The cycle stability of the different samples was tested at a current density of 10 A·g^−1^ as shown in Figure 6b. The capacitance retention ratios of RPM-7.5, RP-7.5 and RM-7.5 after 5000 charge and discharge cycles were 86.1%, 74.1% and 78.2%, respectively. The crystal structure of MnO_2_ of RP-7.5 exhibits good cycle stability. It was found that the cycle stability of RP-7.5 decreased significantly, which may be due to the fact that during the cycle test, the structural strain or volume change caused by ion insertion/extraction reduced its mechanical properties, causing the PANI nanorods to fall off the RCF during the cycle. RPM-7.5 exhibits the best stability, which may be attributed to the ingenious combination of RCF, PANI and MnO_2_. The ternary composite can adjust the volume change caused by ion insertion/extraction to prevent the active material from dissolving and falling off [26].

## 4. Conclusions

In this work, we have successfully prepared RCF (8 μm in diameter) from carbon fiber-reinforced epoxy resin matrix composite through high-temperature pyrolysis. The RCF/PANI/MnO_2_ ternary composite flexible electrode material was fabricated using a one-step electrodeposition technique. The morphological and structural characterization of RPM-7.5 revealed that interconnected PANI nanorods were embedded in MnO_2_ nanoparticles, which increased the contact area between the electrode and the electrolyte, improved the stability of PANI and promoted the diffusion rate of ions and electrons. In addition, the RPM-7.5 composite shows an excellent specific capacitance of 475.1 F·g^−1^ at 1 A·g^−1^ in 1 M Na_2_SO_4_ electrolyte and capacitance retention of 86.1% after 5000 GCD cycles. This high-performance electrode material is prepared on the basis of a simple, green, low-cost method, making it a promising candidate for the preparation of flexible electrodes for supercapacitors.

## Figures and Tables

**Table 1:** Comparison of various carbon/PANI/MnO_2_ composites in electrochemical capacitance performance.

No.	Name	Electrolyte	Specific Capacitance (F·g^−1^)	Scan Rate/Current Density	References
1	MnO_2_/PANI nano-reticulation composites	1 M Na_2_SO_4_	425	0.1 A·g^−1^	[16]
2	MnO_2_@PANI on carbon fiber paper	1 M Na_2_SO_4_	437	1 A·g^−1^	[29]
3	GO/MWCNT/PANI/MnO_2_	0.5 M Na_2_SO_4_	173.00 (mF·cm^−2^)	4 mA·cm^−2^	[31]
4	PANI-assisted graphene@MnO_2_	1 M Na_2_SO_4_	245	0.5 A·g^−1^	[32]
5	AC/MnO_2_/PANI	1 M Na_2_SO_4_	245	1 A·g^−1^	[33]
6	carbon cloth/MnO_2_/PANI	1 M Na_2_SO_4_	1.56 (F·cm^−2^)	10 mV·s^−1^	[34]
7	RPM-7.5	1 M Na_2_SO_4_	475	1 A·g^−1^	This work
